# Supplementary material for: MicroRNA29a Reverts the Activated Hepatic Stellate Cells in the Regression of Hepatic Fibrosis through Regulation of ATPase H+ Transporting V1 Subunit C1
Source: Int J Mol Sci. 2019 Feb 13;20(4):796. doi: 10.3390/ijms20040796 (PMC6412626; doi:10.3390/ijms20040796)
Supplement: Supplementary file 1 [file ijms-20-00796-s001.pdf]

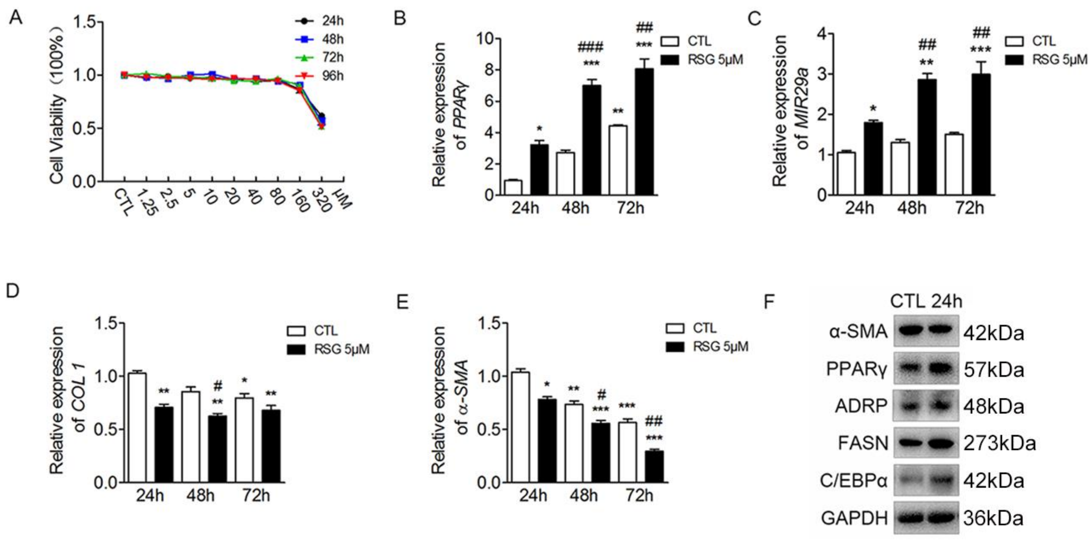

Figure S1. Treatment with rosiglitazone up-regulated expression of *PPARγ* and *MIR29a*. (**A**) The MTT results showed that RSG (0-80 μM) for 24 h, 48 h, 72 h or 96 h had no significant effect on cell viability. (**B**-**E**) RSG was used to treat human HSC cell line LX-2 for 24 h, 48 h and 72 h. The expression of *PPARγ*, *MIR29a*, *COL1* and *α-SMA* were analyzed by qRT-PCR analysis. (**F**) After 24 h RSG (5 μM) treatment, the protein expression of α-SMA, PPARγ, ADRP, FAS, and C/EBPα were analyzed by Western blot in LX-2 cells (n = 2 per group). GAPDH was used as a loading control. Throughout, error bar represents SEM. * P < 0.05, ** P < 0.01 and *** P < 0.001 vs. control (24h) group, # P < 0.05, ## P < 0.01, ###P < 0.001 vs. RSG (24h) group.

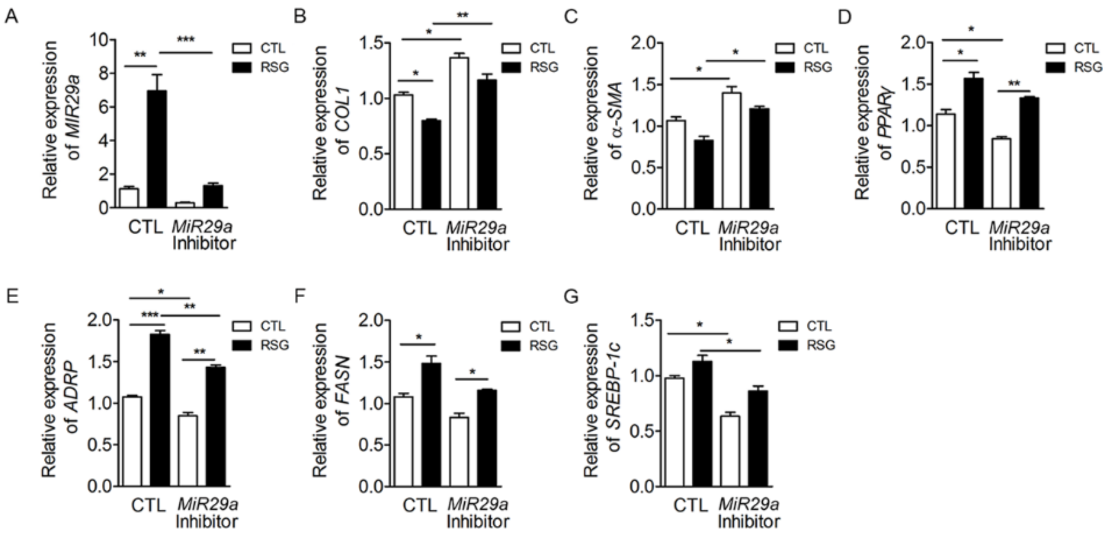

Figure S2. *MIR29a* inhibitor increased the expression of fibrosis-related genes and decreased the expression of adipogenic transcription factors in LX-2 cells. (**A**-**G**) *MIR29a* inhibitor (200 nM) were transiently transfected into LX-2 cells for 48 h, and RSG (5 μM) treated for 24 h, The expression of *MIR29a*, *COL1*, *α-SMA*, *PPARγ*, *ADRP*, *FASN*, and *SREBP-1c* mRNA were assessed by qRT-PCR (n = 3 per group). Throughout, error bar represents SEM. * P < 0.05, ** P < 0.01 and *** P < 0.001.
